# Human Parechovirus Infections in Monkeys with Diarrhea, China

**DOI:** 10.3201/1607.091103

**Published:** 2010-07

**Authors:** T.L. Shan, C.M. Wang, L. Cui, Eric Delwart, C.L. Yuan, W. Zhao, W. Guo, X.Q. Dai, Y. Yu, X.G. Hua

**Affiliations:** Author affiliations: Shanghai Jiao Tong University, Shanghai, People’s Republic of China (T.L. Shan, L. Cui, C.L. Yuan, W. Zhao, W. Guo, X.Q. Dai, Y. Yu, X.G. Hua);; Huazhong Agricultural University, Wuhan, People’s Republic of China (C.M. Wang);; Blood Systems Research Institute, San Francisco, California, USA (E. Delwart);; University of California, San Francisco (E. Delwart); 1 These authors contributed equally to this article.

**Keywords:** Human parechovirus, China, monkey, viruses, zoonoses, dispatch

## Abstract

Information about human parechovirus (HPeV) infection in animals is scant. Using 5′ untranslated region reverse transcription–PCR, we detected HPeV in feces of monkeys with diarrhea and sequenced the complete genome of 1 isolate (SH6). Monkeys may serve as reservoirs for zoonotic HPeV transmissions and as models for studies of HPeV pathogenesis.

Members of the *human parechovirus* (HPeV) species are small, nonenveloped RNA viruses that are members of the family *Picornaviridae,* genus *Parechovirus*. HPeV can be classified into at least 8 genotypes on the basis of sequence similarity of their capsid protein (HPeV-1–HPeV-8). HPeV-1 and HPeV-2, formerly known as echovirus 22 and echovirus 23, were originally considered to belong to the genus *Enterovirus* (*1**,**2*) but after genome sequencing were reclassified as members of a new genus in the family *Picornaviridae* (*3*). Recently, 6 other genotypes of parechovirus were isolated from young children with gastrointestinal, respiratory, and severe neurologic signs (*4**–**11*). Other HPeV genotypes continue to be characterized (www.picornaviridae.com/parechovirus/hpev/hpev.htm).

Despite the frequent infections and numerous HPeV genotypes detected in humans, information about HPeV infection in animals is scant. In this study, we detected HPeV in feces of monkeys with diarrhea and sequenced the complete genome of 1 isolate (SH6).

## The Study

In April 2008, fecal specimens were collected from 116 macaques (3–6 years of age) with diarrhea on a monkey farm in People’s Republic of China. Feces were suspended to 10% (wt/vol) in phosphate-buffered saline (0.01 M, pH 7.4), and total RNA was extracted from 200 µL by using TRIZOL reagent (Invitrogen, Carlsbad, CA, USA). Viral RNA was dissolved in 30 µL RNase-free water and stored at –80°C.

Primers (outside-L 5′-CTAGAGAGCTTGGCCGTCGG-3′, outside-R 5′-GTACCTTCTGGGCATCCTTC-3′, inside-L 5′-GGCCTTATACCCCGACTTGC-3′, and inside-R 5′-GGCCTTACAACTAGTGTTTG-3′) (*12*) were used for reverse transcription nested PCR to identify diverse HPeV genotypes by amplification of a 518-bp fragment located in the 5′ untranslated region (UTR). The expected-size DNA bands were excised from an agarose gel, purified with the AxyPrep DNA gel extraction kit (Axygen, Union City, CA, USA), cloned into pMD-18T vector (TaKaRa, Dalian, China), and sequenced (Applied Biosystems 3730 DNA Analyzer; Invitrogen). Feces from 6 of 116 monkeys were positive for HPeV. The HPeV sequences were compared with those of the HPeV genotype reference strains by using BLAST (www.ncbi.nlm.nih.gov/BLAST). Five of the 6 sequences showed closest identity to the 5′ UTR of HPeV-1 (90%–94%). The viral protein (VP)3/VP1 region of these 5 viruses was then PCR amplified and sequenced to confirm type 1 identity (*13*). The last monkey feces–derived sequence showed 97%–98% nucleotide identity to HPeV-6 strains. This strain was fully sequenced.

A 674-nt region of the 5′ UTR, an open reading frame (ORF) encoding a polyprotein precursor of 2,182 aa, and a partial 3′ UTR of 88 nt (7,311 bp) were sequenced. The near full genome showed 96% nucleotide identity with the genotype 6 reference genome (AB252582). The polyprotein encoded capsid proteins VP0 (312 aa), VP3 (229 aa), and VP1 (234 aa) and nonstructural proteins 2A (150 aa), 2B (122 aa), 2C (329 aa), 3A (117 aa), 3B (20 aa), 3C (200 aa), and 3D (469 aa). The integrin binding motif arginine–glycine–aspartic acid was identified close to the C terminus of VP1 (*8**,**14*).

We performed phylogenetic analysis using the nearly full genome of SH6 and 17 representative HPeV and related viruses (Figure). Results confirmed that SH6 belonged to genotype 6 and clustered closely with the reference genome from Japan and a strain from the Netherlands (EU077518), forming an HPeV-6 subgroup (Figure).

**Figure:**
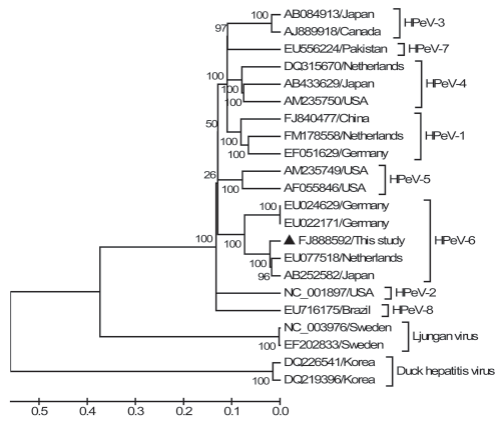
Phylogenetic analysis of the complete genomes. Phylogenetic tree was constructed by the neighbor-joining method with 1,000 bootstrap replicates using MEGA4.0 software (www.megasoftware.net) with an alignment of the nearly full genome isolated in this study and 17 human parechovirus (HPeV) and related genomes. Bootstrap values are indicated at each branching point. GenBank accession numbers and countries of origins are indicated. The isolate of genotype 6 is marked with a triangle. Scale bar indicates estimated phylogenetic divergence.

## Conclusions

Previous studies have documented frequent human infections with different HPeV genotypes (www.picornaviridae.com/parechovirus/hpev/hpev.htm). We detected HPeV genotypes 1 and 6 in the feces of 6 of 116 monkeys with diarrhea. A similar analysis of a healthy monkey control group is needed to determine whether an association exists between HPeV infections of monkeys and diarrhea. On the basis of the close similarities between virus feces–derived sequences and HPeV, these viruses might have been transmitted by the fecal–oral route from humans to monkeys. The multiple HPeV genotypes detected indicated that viral transmission occurred on multiple occasions. Monkeys may therefore serve as an animal model for HPeV infection and possibly pathogenesis.
